# Adult-Onset Mitochondrial Encephalopathy, Lactic Acidosis, and Stroke-Like Episodes (MELAS) in a Patient Without Significant Family History

**DOI:** 10.7759/cureus.21597

**Published:** 2022-01-25

**Authors:** Shiwani Kamath, Neel A Duggal, Abid Ulhaque, Elliott Taylor, Parth Desai

**Affiliations:** 1 Internal Medicine, Medical Center of Trinity, Trinity, USA; 2 Radiology, Medical Center of Trinity, Trinity, USA; 3 Critical Care Medicine, Medical Center of Trinity, Trinity, USA

**Keywords:** diagnosis of rare cases, recurrent seizures, stroke-like symptoms, mitochondrial disorder, melas

## Abstract

This case reports a 53-year-old Caucasian female previously diagnosed with viral encephalitis and Fahr's Syndrome who presented with altered mental status. Shortly after arrival, she displayed severe lactic acidosis and was transferred to the intensive care unit (ICU), where she had a brief seizure. Neurological workup was performed including carotid ultrasound, magnetic resonance angiography (MRA) brain, and computed tomography (CT) angiogram of the neck, all of which were unremarkable. Initial magnetic resonance imaging (MRI) performed showed small, acute ischemic foci in the bilateral occipital lobes and medial left thalamus. Subsequent diffusion-weighted imaging (DWI) MRI of the bilateral occipital lobes showed vasogenic edema, a common finding in Mitochondrial Encephalopathy, Lactic Acid, and Stroke-like episodes (MELAS). The patient was given Levetiracetam and managed supportively. She was progressively extubated and her seizure symptoms and lactic acidosis resolved. Our case represents a unique case in which a patient with non-contributory family history is first diagnosed with MELAS after age 40 after her symptoms were initially attributed to other pathologies.

## Introduction

Mitochondrial encephalopathy, lactic acidosis, and stroke-like episodes (MELAS) is a mitochondrial disorder with multi-organ manifestations that occurs in one out of 4000 individuals [1-2]. It usually presents early in life in children and young adults and has an unrelenting disease course. MELAS is defined by stroke-like episodes characterized by hemiparesis or visual changes. Other common clinical manifestations include seizures, encephalopathy, dementia, epilepsy, hearing impairment, short stature, and recurrent headaches [1-2]. It is often associated with diabetes mellitus and cardiac conduction disorders [2]. Due to its variable presentation, the diagnosis of MELAS is frequently overlooked and often missed. In this case report, we present a case of MELAS previously diagnosed as viral encephalitis, and later, as recurrent strokes.

## Case presentation

A 55-year-old female with a past medical history of stroke-like symptoms and seizures with a diagnosis of Fahr's Syndrome, meningoencephalitis, chronic obstructive pulmonary disease (COPD), and hyperlipidemia presented to the emergency room from her assisted living facility with altered mental status. At baseline, the patient used a walker. She notified her caretakers earlier that morning that she had back pain, felt generally unwell, and wanted to go to the hospital. When emergency services arrived, she was awake and able to move extremities; however, she appeared distressed, was non-verbal, and did not follow commands. The patient's caretakers stated that she was not taking any prescribed medications at home. 

Upon admission to our emergency department, the patient’s vitals were stable. On physical examination, she was non-verbal and did not follow commands, but she was able to move extremities and open eyes spontaneously. Pupils were 4 mm and non-reactive. Due to a history of seizures and concern for a possible post-ictal phase, she was given stat intravenous (IV) levetiracetam, 1000 mg. Tele-neurology was consulted and the patient was sent for CT head imaging. 

**Figure 1:**
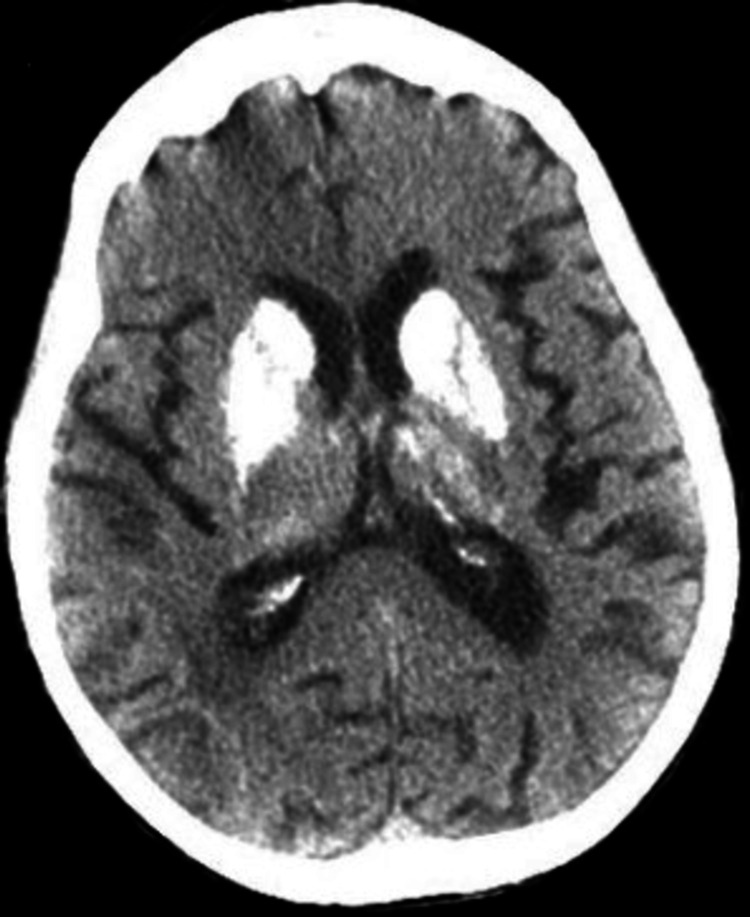
Non-Contrast CT of the brain, axial view This image shows bilateral calcification of the basal ganglia.

**Figure 2:**
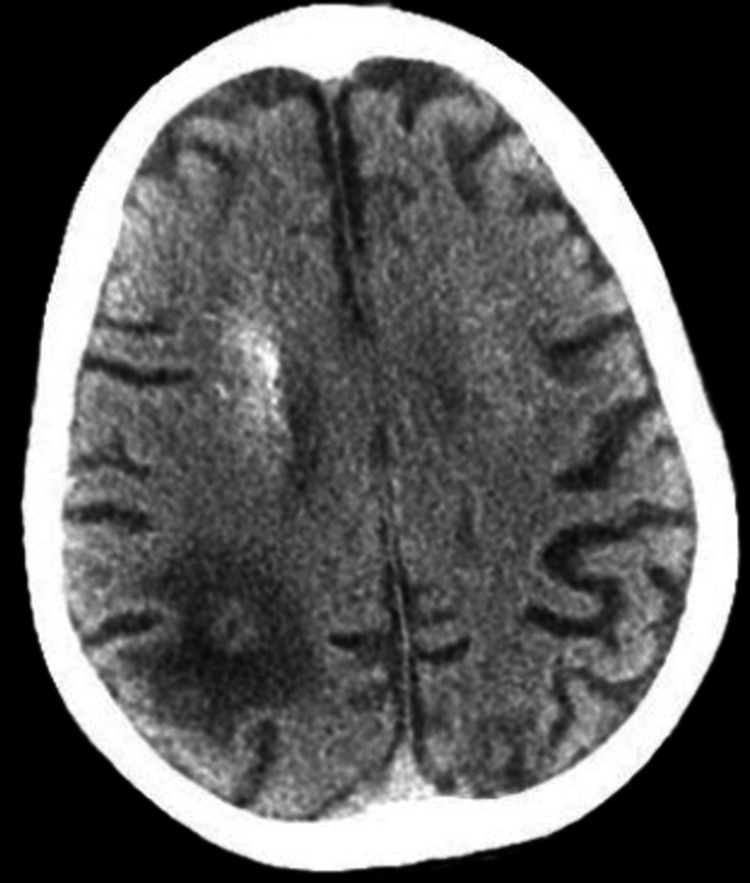
Non-Contrast CT of the brain, axial view This image demonstrates a large hypodense lesion at the right occipito-parietal lobe, showing an old infarct.

On return from CT, the patient was noted to have slow, shallow respirations. Arterial blood gas (ABG) findings (Table 1) are indicative of severe acidosis, and the patient was subsequently intubated. On review of her laboratory studies, blood glucose was elevated to 343 mg/dL and lactic acid was elevated to 11.4 mmol/L. Corresponding Sodium ion (Na^+)^ was 136 and Chloride ion (Cl^- ) ^104, with a calculated anion gap of 28.9, indicating the presence of anion gap metabolic acidosis. In the setting of metabolic acidosis with uncompensated respiratory alkalosis, we suspected possible diabetic ketoacidosis or an elevated lactic acid due to seizures or sepsis. Blood pressures were low at 84/60 mmHg, and after insufficient response to fluid bolus, a norepinephrine drip was initiated. Urine showed trace ketones (5 mg/dl), and insulin drip protocol was ordered for diabetic ketoacidosis (DKA). Empiric IV vancomycin and ceftriaxone for septic shock. At this time, CT head imaging returned with an old posterior parietal infarct and extensive bilateral calcifications suspicious for Fahr's Syndrome, as seen in Figures 1, 2.

**Table 1:** Arterial blood gas findings pH: potential of hydrogen, pCO2: partial pressure of carbon dioxide, pO2: partial pressure of oxygen

Arterial Blood Gas	Result	Normal Range
pH	6.92	7.350-7.450
pCO2 (mmHg)	15.6	35.0-45.0
pO2 (mmHg)	159	80.0-105.0
Bicarbonate (mEq/L)	3.1	22.0-26.0

**Figure 3:**
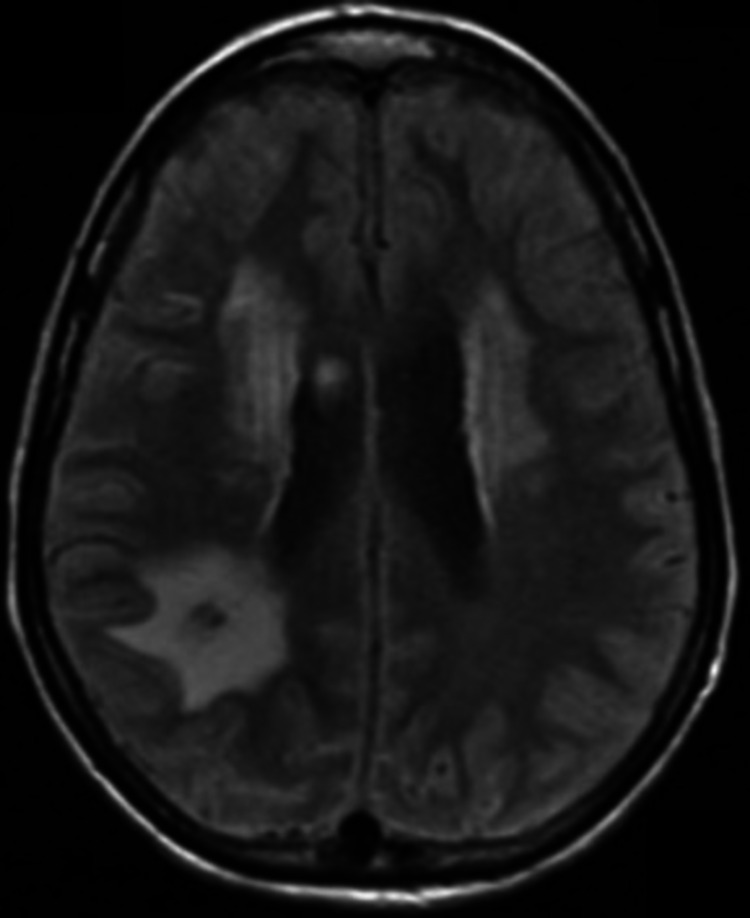
MRI Brain, axial view This image shows an axial view of the patient’s MRI Brain showing an old infarct in the right occipito-parietal junction consistent with CT findings. There are periventricular hyperintense lesions in the subcortical white matter tracts showing chronic small ischemic disease. The small vessel disease is uncommon in patients in this age group but is explained by the underlying mitochondrial disorder.

**Figure 4:**
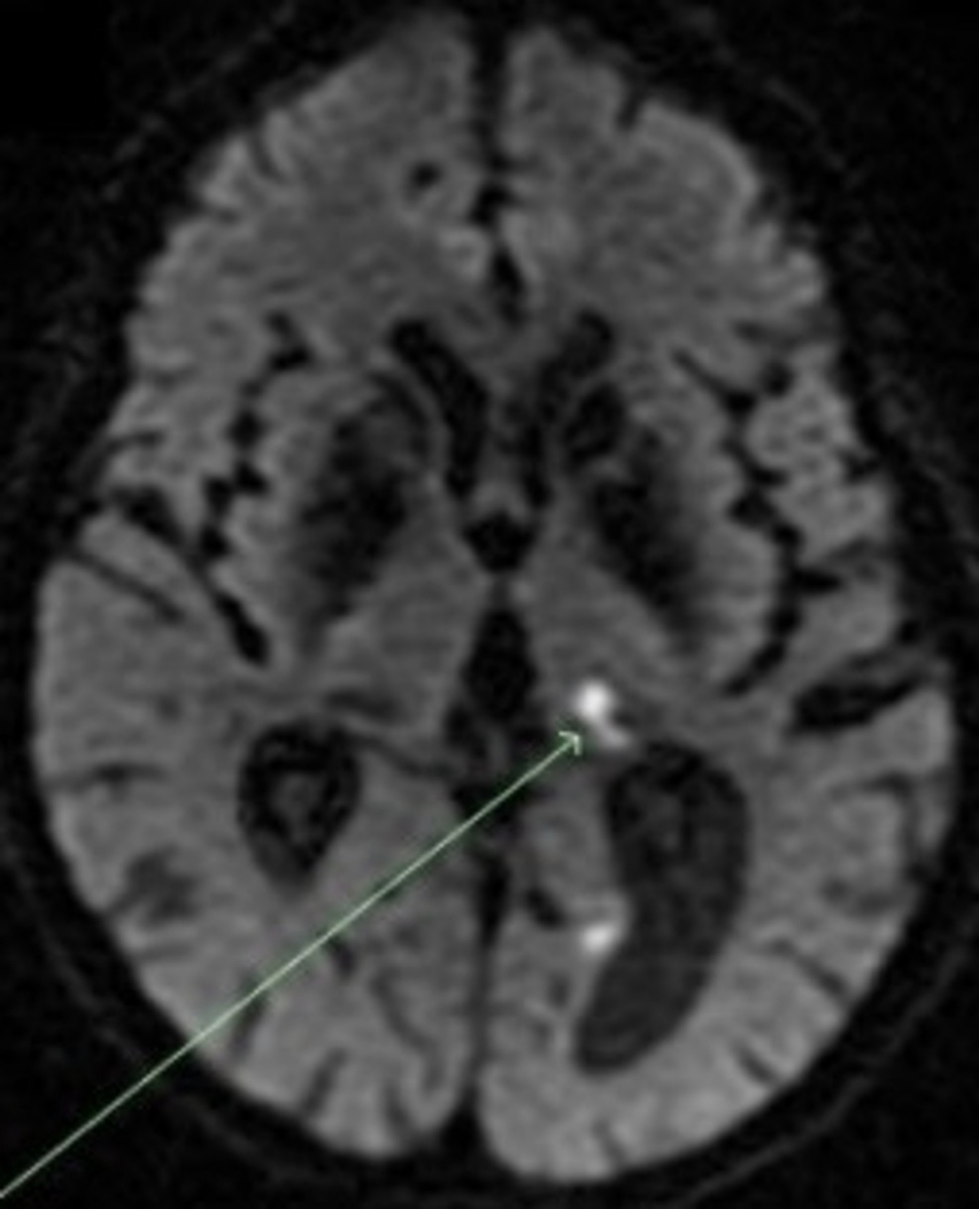
DWI sequence, axial view This image depicts an axial view of a DWI sequence that shows restricted diffusion in the left occipital lobe and in the medial left thalamus (indicated by the green arrow).

**Figure 5:**
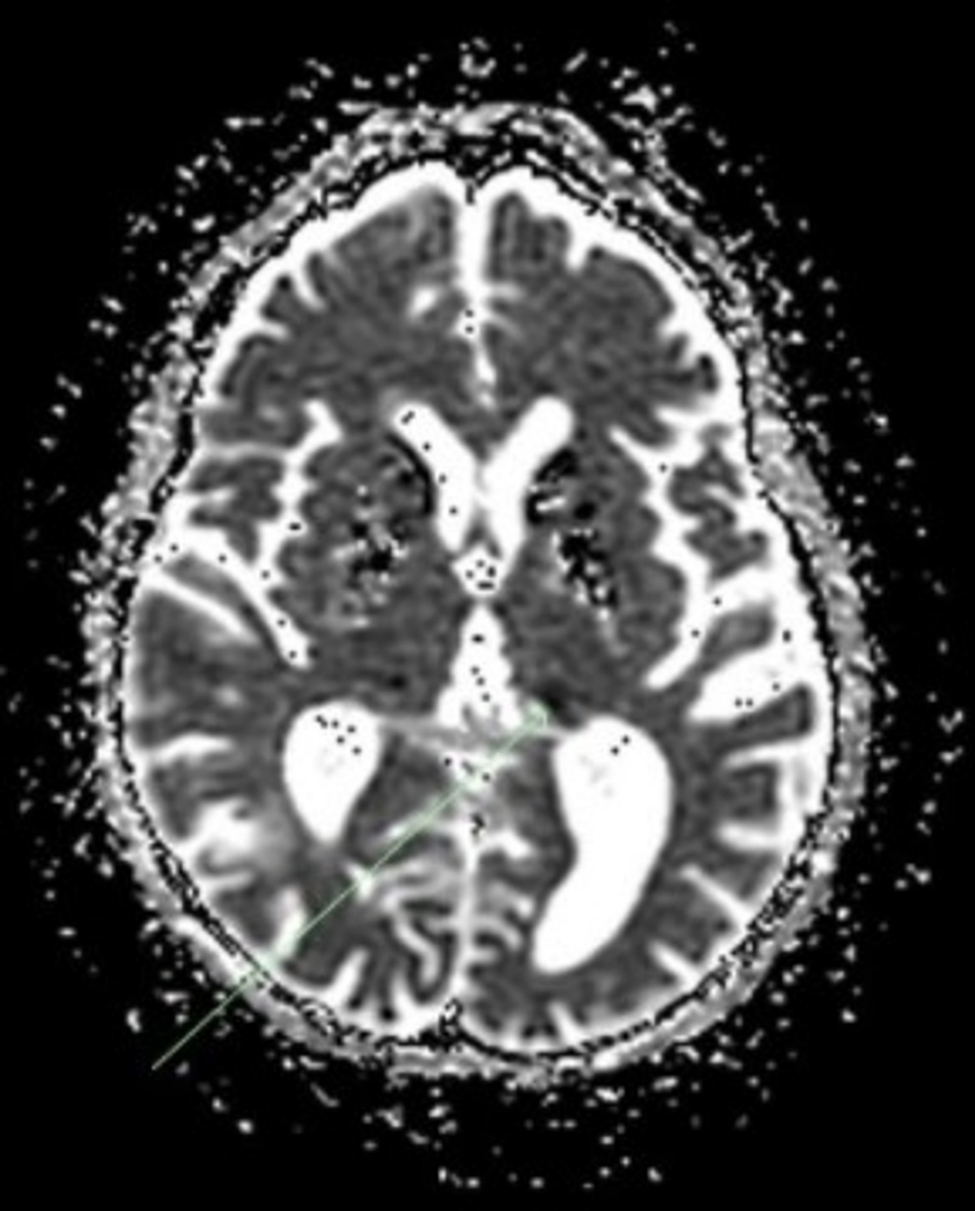
Axial view of the ADC map This image shows an axial view of the apparent diffusion coefficient (ADC) map at the same level, showing a hypointense lesion in the medial left thalamus. This finding supports an acute thrombotic event.

On arrival to the intensive care unit (ICU), the patient was sedated with stat IV Ativan 4 mg to Richmond agitation sedation scale of -2. She then experienced a generalized tonic-clonic seizure that self-resolved within approximately one minute. Magnetic resonance imaging (MRI) performed showed small acute ischemic foci in the bilateral occipital lobes and medial left thalamus (as seen in Figures 3, 4, 5). Neurology was consulted, who initiated treatment with atorvastatin and acetylsalicylic acid for acute stroke management and Levetiracetam for seizures. They performed workup including carotid ultrasound, MRA brain, and CT angiogram of the neck, all of which turned out negative. 

The patient’s past medical history was then obtained from her family. Per the patient’s family, she started having hearing difficulties and forgetfulness 16 years prior to this admission. Eleven years prior, the patient presented with similar neurological symptoms as well as seizures, and she was subsequently admitted to the ICU and intubated. CT imaging at that time showed diffuse intracranial calcification suggestive of Fahr’s syndrome. On discharge, the patient had ataxia, but she was otherwise able to do activities of daily living and only required support with financial and legal decisions.

At this time, we suspected that the patient may have MELAS. This was due to the patient’s persistent encephalopathy and non-focal neurological findings, which were unexplained by the stroke or a postictal state, as well as her lactic acidosis. Imaging was thoroughly reviewed with the radiology team, who suggested that the comparison of images on the MRI after DWI was indicative of MELAS (Figure 4). The diffusion restriction in the bilateral occipital lobes (the right lobe is not seen in these images visualized in Figure 5) did not have the corresponding hypoattenuation on the apparent diffusion coefficient (ADC) map. These findings cumulatively represent the T2 shine-through phenomenon and show vasogenic edema, a common finding in MELAS syndrome. 

The patient’s family deferred cerebrospinal fluid (CSF) examination and expressed preference for genetic and muscle biopsy testing to be pursued in an outpatient setting. The patient’s alertness and neurological participation improved, and she was extubated. Her lactic acid continued to downtrend and the source of infection was ruled out. She was downgraded from ICU to the hospitalist service where her neurological and physical status continued to approach her baseline. The patient was free of seizures throughout the rest of her hospital course and was transferred to a skilled nursing facility. 

The patient was readmitted one month later with small bowel obstruction, as well as acute encephalopathy and lactic acidosis secondary to MELAS. Lactic acid was 10.6 mmol/L. Further testing and imaging did not provide further information. With supportive care, the patient reached her baseline and lactic acid resolved. The patient was discharged with instructions to undergo genetic testing and muscle biopsy as an outpatient, which was not completed.

## Discussion

Our case presentation chronicles a case of a patient with a history of encephalopathy and seizures previously diagnosed with Fahr's syndrome. Throughout her stay, she had hyperglycemia and CT imaging showed basal ganglia calcification and right posterior parietal infarct. While she and her family were instructed to obtain genetic testing at discharge to further support the diagnosis of MELAS, this was ultimately not obtained to our knowledge. Mitochondrial diseases are very heterogenous due to heteroplasmy, and they often have diabetes regardless of having the MELAS phenotype or not. Basal ganglia calcification is the most common imaging finding in A3243G mutation.

MELAS is typically inherited maternally, but the patient’s family in our case denied any known family history or manifestations of MELAS such as seizures or stroke-like symptoms in her mother.Rarely, however, MELAS may occur due to a sporadic mutation in a patient without a family history of it [2]. Regardless of etiology, symptoms typically first appear by the age of 40 years [2].

Several recent case reports have documented patients with no family history of MELAS that were diagnosed after the age of 50 [3-6]. These cases show wide variability in clinical presentation. In a report similar to ours, Yokota et al. documented a 73-year-old man with no known relevant family history presenting with a stroke-like episode characterized by altered mental status and an afebrile seizure that was initially attributed to herpes simplex virus (HSV) encephalitis [3]. As our patient, after DWI imaging showed lesions in the occipital areas and elevated lactate in the CSF, a diagnosis of MELAS was considered and confirmed with genetic testing [4]. Cases of MELAS first diagnosed in patients older than 50 years with a contributory family history have also been reported [7].

The 2012 Japanese diagnostic criteria are typically used to diagnose MELAS [8]. For a definitive diagnosis, individuals must meet at least two criteria in two sets of categories. Category A includes headaches with vomiting, seizures, hemiplegia, cortical blindness, and acute focal findings on neuroimaging. Criteria B includes high plasma/CSF lactate, mitochondrial abnormalities on muscle biopsy, or a MELAS-related pathogenic variant. Our patient exhibited seizures, acute focal findings, and high plasma lactate. However, we were unable to perform a genetic analysis or muscle biopsy while hospitalized due to the preferences of the patient and her family, and this was not obtained in the outpatient setting.

MELAS is an uncommon diagnosis and one not often considered in patients, especially in older patients with no relevant family history. Per patient history obtained from the family, our patient exhibited clinical features suspicious for MELAS, including seizures, stroke-like episodes, and elevated lactic acidosis. However, the diagnosis of MELAS was not considered by clinicians who saw the patient, and she did not undergo any confirmatory testing in the past decade. When these clinical features appear frequently in patients without family history and other plausible explanations, clinicians should consider MELAS as a possible explanation in patients. With an earlier diagnosis, more appropriate management can be done which potentially improves patient quality of life.

Imaging is important in evaluating possible MELAS. DWI and ADC help distinguish between stroke and stroke-like episodes. In ischemic parts of the brain, diffusion is typically restricted due to cytotoxic edema, causing a decrease in signal intensity on the ADC map. In MELAS, lesions are typically caused by vasogenic edema, and the signal intensity is either the same or not as reduced compared to DWI [9]. This is sometimes referred to as T2 shine through, which was noted in images of our patient. Basal Ganglia calcification is another prominent feature seen in older patients with MELAS* *[10]. It is also common in Fahr's Syndrome, but our patient's corresponding lactic acidosis and stroke-like manifestations make MELAS a more compelling diagnosis.

There are currently no consensus guidelines in managing MELAS [11, 12]. Treatment of MELAS, like in our case, is typically interdisciplinary and supportive, with no current medications known to prevent the progression of the disease. L-arginine is thought to improve stroke-like episodes and decrease the frequency and severity of these episodes, while L-citrulline is thought to prevent stroke risk [13]. These drugs are theorized to work by increasing nitric oxide (NO), as arginine and citrulline are precursors of NO. Carnitine and coenzyme Q10 are commonly used without proven efficacy from controlled trials; however, they are thought to increase energy production from the mitochondria [1-2]. Because stroke-like symptoms are sometimes triggered by seizures, anticonvulsants and high-dose glucocorticoids may play a role in management, as used in our patient. 

## Conclusions

MELAS is a very rare cause of stroke-like events and seizures. Its diagnosis is often overlooked, especially in patients over the age of 40 or in patients without a known family history. This diagnosis should certainly be considered when evaluating patients with recurrent stroke or stroke-like events that have MRI findings uncharacteristic of ischemic stroke, especially those patients exhibiting the aforementioned clinical symptoms and lactic acidosis. 
